# Adsorptive and Reductive Removal of Chlorophenol from Wastewater by Biomass-Derived Mesoporous Carbon-Supported Sulfide Nanoscale Zerovalent Iron

**DOI:** 10.3390/nano9121786

**Published:** 2019-12-16

**Authors:** Hui Wang, Sixiang Cai, Liang Shan, Min Zhuang, Nan Li, Guixiang Quan, Jinlong Yan

**Affiliations:** 1 School of Environmental Science and Engineering, Key Laboratory for Advanced Technology in Environmental Protection of Jiangsu Province, Yancheng Institute of Technology, Yancheng 224051, China; whsl@ycit.cn (H.W.); z17802592726@163.com (M.Z.); ln_9597@163.com (N.L.); qgx@ycit.cn (G.Q.); 2 State Key Laboratory of Marine Resource Utilization in South China Sea, Hainan University, Haikou 570228, China; 3 Yancheng Environmental Engineering Technology Research and Development Center, School of Environment, Tsinghua University, Yancheng 224051, China; shanliangsir@126.com

**Keywords:** porous carbon, zerovalent iron, biomass, dichlorination, adsorption

## Abstract

Chlorinated compounds in a water environment pose serious threats to humanity. A nanoscale zerovalent iron (nZVI) has desirable properties for water dichlorination, but its reactivity is still limited by agglomeration and oxidation. In this study, the mesoporous carbon (MC) derived from biomass waste was prepared for immobilizing nZVI, and the nZVI@MC was further modified by sulfur (S-nZVI@MC) to relieve surface oxidation. The synergistic effect between nZVI and surface modification, the reaction conditions and the removal mechanism were investigated systematically. The characterization results showed nZVI was successfully loaded on the surface of MC, and the aggregation of nZVI was prevented. Moreover, sulfidation modification resulted in the formation of FeS on the surface of nZVI, which effectively alleviated surface oxidation of nZVI and promoted the electron transfer. Batch experiments demonstrated S-nZVI@MC had greatly enhanced reactivity towards 2,4,6-trichlorphenol (TCP) as compared to MC and nZVI, and the removal rate could reach 100%, which was mainly attributed to the significant synergistic effect of MC immobilization and sulfidation modification. Furthermore, the TCP removal process was well described by a Langmuir adsorption model and pseudo-second-order model. The possible mechanism for enhanced removal of TCP is the fast adsorption onto S-nZVI@MC and effective reduction by S-nZVI. Therefore, with excellent reducing activity and antioxidation, S-nZVI@MC has the potential as a pollutant treatment.

## 1. Introduction

Nowadays, the scarcity of water resources and increasing water contamination are a worldwide environmental problem that have drawn extensive attention. Chlorophenols are a class of typical organic contaminants that have been utilized widely in various industrial and agricultural fields. Specifically, 2,4,6-trichlorphenol (TCP) has been listed among United States Environmental Protection Agency (US EPA) priority pollutants due to its high toxicity, mutagenicity, and carcinogenicity [1,2]. Various methods such as photodegradation, chemical reduction and adsorption have been developed to remove chlorophenols from contaminated water [3,4,5]. However, the methods usually involve high operating cost and complicated operation processes, which limit their practical application. Hence, it is highly desirable to develop a cost-effective method to remove TCP from contaminated water.

In the past two decades, nanoscale zerovalent iron (nZVI) has been demonstrated to be an effective solution for the degradation/elimination of various pollutants including chlorinated organic compounds, heavy metal ions and antibiotics due to its strong reductive capability, low cost and environmental friendliness [6,7,8]. However, nZVI particles are easily agglomerated due to their high surface energy and intrinsic magnetic interaction, resulting in the rapid deactivation of nZVI in the operation process [9,10]. Moreover, nZVI particles are readily oxidized under ambient environmental conditions [11]. The above drawbacks limit the environmental application of nZVI.

Immobilizing nZVI particles on porous material supports has been demonstrated as a tactful way to obtain high-efficiency and stable nZVI [12]. On the one hand, porous supports have a certain adsorption capacity for the pollutants, thereby improving the removal efficiency. On the other hand, porous supports can provide stable sites for nZVI particles, and thus prevent the agglomeration of the nanoparticles. Compared with various porous materials, carbon supports have attracted great attention due to the large specific surface area, porous structure and good chemical stability [13,14]. For instance, Wu et al. found that the removal efficiency of bromate by nZVI/activated carbon can be improved due to its excellent adsorption performance [15]. Although the traditional activated carbon has a high specific surface area, micropores predominate in its structure, which is not conducive to the effective dispersion of nZVI, and the diffusion resistance also limits the transport of pollutant molecules. Therefore, mesoporous carbon has been applied for the nZVI support owing to the large surface area, the promotion of target pollutant diffusion as well as the excellent nZVI immobilization abilities provided by the mesoporous channels. Ling et al. found that loading nZVI on a mesoporous carbon significantly increased its reactivity towards nitrobenzene [16]. The mesoporous carbon is usually prepared through a templating method using mesoporous silica as hard templates or amphiphilic copolymer surfactants as soft templates and resin as carbon source. However, such fabricating approaches usually involve complicated and costly preparation procedures, which have limited their practical applications. Therefore, it is still challenging to synthesize mesoporous carbon-supported nZVI through a facile and economical method.

It should be noted that although supported nZVI can prevent the agglomeration and improve the stability of nZVI, it is easily oxidized by water, O_2_ and other coexisting solutes, which would impede electron transfer from nZVI core to target contaminates [17]. In view of the above issue, recent research has found that sulfidation of nZVI is the most promising approach to suppress surface oxidation and increase reactivity of nZVI [18,19,20]. On the one hand, the iron sulfide shell on the surface of nZVI acts as a barrier to prevent nZVI contacting water and mitigating the its surface corrosion. On the other hand, the iron sulfide has higher electronegativity (5.02 V) than nZVI (4.04 V), which can facilitate the rapid transfer of electrons. For instance, Gao et al. found the reactivity of iron sulfide coated iron nanoparticles towards Cr(VI) was significantly better than bare nZVI [21]. He et al. demonstrated that the sulfidation can improve the dichlorination rate and selectivity of nZVI [22].

**Figure 1:**
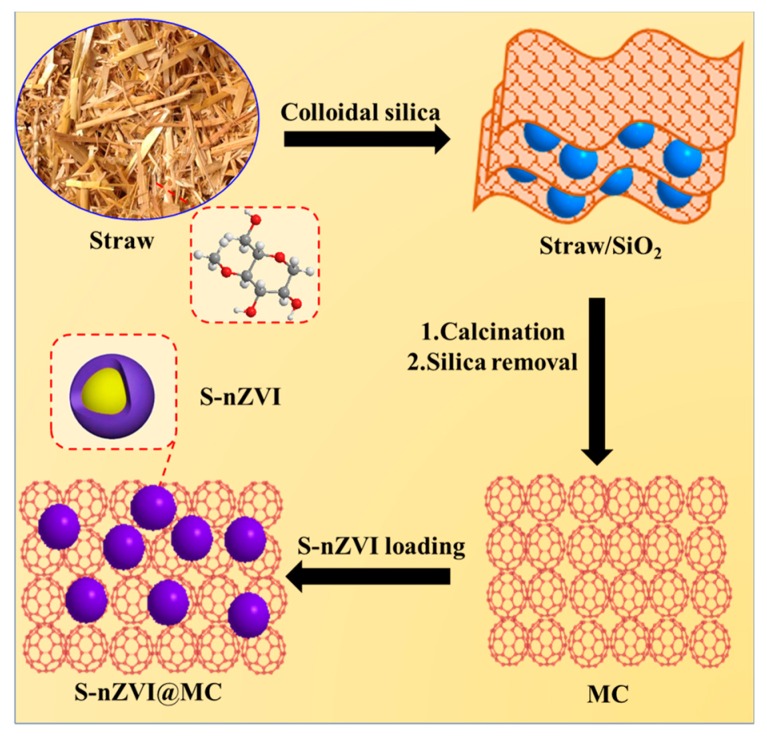
Schematic illustration of the sulfidized nanoscale zerovalent iron supported on mesoporous carbon (S-nZVI@MC) formation.

To take advantage of the recent development in nZVI, a sulfidized nZVI supported on MC (S-nZVI@MC) was prepared and used to remove TCP from aqueous solutions. As seen from Figure 1, the MC was synthesized simply using straw waste as carbon source and colloidal silica as hard template, which simplified fabricating processes and reduced synthesis cost. With MC as a supporter, the S-nZVI was successfully loaded on its surface by reduction of Fe (III) ions with NaBH_4_ in the presence of dithionite. The aggregation and surface oxidation of nZVI has been effectively alleviated due to the synergistic effect of loading and sulfidation, which is beneficial to improve its reactivity and life. The S-nZVI@MC was further used for TCP removal from water and the effects of solution pH, TCP initial concentration, S-nZVI@MC dosage were examined. The finding of this study paves the way to developing high-performance and low-cost nZVI for dichlorination and reduction of other pollutants.

## 2. Materials and Methods

### 2.1. Synthesis of S-nZVI@MC

Materials: wheat straw was obtained from Yancheng, Jiangsu, P. R. of China. To remove impurities, the wheat straw is fully washed with deionized water and anhydrous ethanol before use. 2,4,6-Trichlorophenol (TCP, >98%) was purchased from Aladding Chemistry Co. Ltd., Shanghai, China. Hydrofluoric acid (HF, AR) was obtained from Sinopharm Chemical Reagent Co., Ltd., Shanghai, China. Milli-Q water (18.25 MΩ cm^−1^) was used throughout the experiment.

Per-carbonization of the wheat straw: in typical synthesis, wheat straw (3.0 g) was added into the H_2_SO_4_ solution (100 mL, 5%, V:V), and then stirred for 15 min. The above solution was transferred into the Teflon vessel and reacted for 12 h at 180 °C. After cooling to room temperature, the product was washed by deionized water until reaching a pH of 7, and then dried at 80 °C in a conventional oven. Finally, the per-carbonized wheat straw was obtained.

Synthesis of MC: typically, the per-carbonized wheat straw was soaked in 1.0 wt % colloidal silica aqueous solution, and mass ratio of silica/straw was 3:10. The above solution was evaporated at 85 °C through continuous stirring until becoming a viscous solution. After drying at 80 °C in a conventional oven, a solid powder was formed. To obtain the MC, the precursor was calcinated at 800 °C for 2 h under a N_2_ atmosphere with the ramp rate of 3 °C/min. After HF etching and water wash, the silica can be removed and MC was finally formed.

Synthesis of S-nZVI@MC: 0.2 g MC was firstly added into 100 mL DI and ethanol mixture solution (V:V = 7:3). After sonicating for 60–120 min, the above mixture solution was poured into a 250 mL three-necked bottom flask equipped with a mechanical stirrer, and a thermometer with a temperature controller and a N_2_ inlet; 1.15 g of FeSO_4_·7H_2_O was then added to the three-neck flask, and after being stirred for about 20 min in N_2_ atmosphere, 100 mL containing 7.6 g sodium borohydride and different amounts of Na_2_S_2_O_6_ (samples here referred as S-nZVI@MC-0.05, S-nZVI@MC-0.125, S-nZVI@MC-0.25, whose S/Fe molar ratio were approximate 0.05, 0.125 and 0.25, respectively) were added in a dropwise manner to the above FeSO_4_ solution. After the injection, stirring continued for 30 min to ensure adequate reaction. S-nZVI@MC were harvested by centrifugation and washed with ethanol three times and then dried in a vacuum. The obtained samples were carefully stored in the glove box filled with inert gas.

### 2.2. Characterization

The detailed morphological information of the samples was further analyzed by transmission electron microscopy (TEM, JEOL JEM-200CX, Tokyo, Japan) and scanning electron microscopy (SEM, JEOL JEM-6700F, Tokyo, Japan) tests. Before the TEM testing, the samples were dispersed in ethanol via ultrasonication for 15 min, and then deposited a few drops onto a carbon-coated copper grid. X-ray diffraction (XRD, Rigaku Corporation, Tokyo, Japan) measurements were recorded by the X-ray diffractometerusing Cu Kα radiation (40 kV, 3020 mA). X-ray photoelectron spectroscopy (XPS, Perkin–Elmer, Hopkinton, MA, USA) was tested on a Perkin-Elmer PHI 5000C ESCA (Waltham, MA United States) system with a dual X-ray source, using the 45 MgKα (1253.6 eV) anode and a hemispherical energy analyser. Before all tests, the samples were sealed into sample tubes in a glove box, and then shipped to the test instrument.

### 2.3. Adsorptive and Reductive Performance

Batch adsorption experiments were conducted by shaking a mixture of obtained S-nZVI@MC with 100 mL TCP aqueous solution of certain concentration in a series of 150 mL plastic bottles. Such adsorption experiment parameters as contact time (0–180 min), initial concentration of TCP (30–100 mg L^−1^) and solution pH (4–11) were further investigated. The solution pH was adjusted by 0.1 M HCl or NaOH solution. The solid-liquid phases were taken out after shaking for a predetermined time, and followed by filtration using 0.22 μm cellulose nitrate membrane filter. The concentration of TCP was measured with an ultraviolet–visible spectrophotometer (TU 1810, Beijing Purkinje General Instrument Co. Ltd., Beijing, China). The removal efficiency was calculated according to the following equations:

*η* = (C_0_ − C_t_)/C_0_ (1)

where C_0_ and C_t_ (mg L^−1^) are the initial and final concentrations of TCP in the feed solution, respectively.

## 3. Results

### 3.1. Characterization

**Figure 2:**
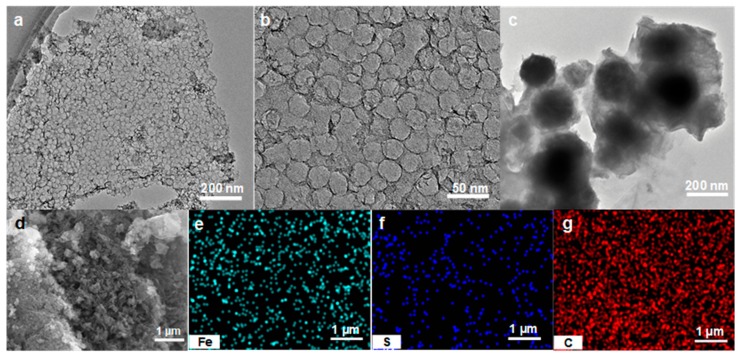
(**a**,**b**) Transmission electron microscope (TEM) images of mesoporous carbon (MC), (**c**) TEM image, (**d**) scanning electron microscope (SEM) image and (**e**–**g**) elemental mapping images of S-nZVI@MC.

The morphology of the MC and S-nZVI@MC was firstly investigated by TEM and SEM. As presented in Figure 2a,b the MC shows a well-formed porous structure, and the pore size is about 22 nm, which is consistent with the size of the silica template. The mesoporous structure not only has a high specific surface area for nZVI particle dispersion, but also provides a large number of smooth channels for the TCP molecules diffusion. As shown in Figure 2c, the MC surface is covered with flake-like structure, indicating that the S-nZVI was successfully loaded on the MC. The S-nZVI has a core-shell structure, and the Fe^0^ core is loosely surrounded by FeS. The agglomeration of nZVI is obviously relieved, indicating the MC supporter can anchor nZVI and promote its dispersion. In addition, the FeS shell can also effectively reduce the magnetic attraction between nZVI particles and prevent its re-agglomeration. As seen from the SEM image of S-nZVI@MC in Figure 2d, the discrete S-nZVI with globular structure covered the surface of MC. Moreover, with elemental energy dispersive spectrometer (EDS)mapping shows a distribution of Fe, S, and C in S-nZVI @MC (Figure 2e–g). The notable Fe and S distribution confirmed the presence of Fe and S after the nZVI loading and sulfide modification. Therefore, combined with loading and sulfidation, the agglomeration of nZVI was effectively inhibited, which is beneficial to active site exposure, and thus promotes its reactivity.

**Figure 3:**
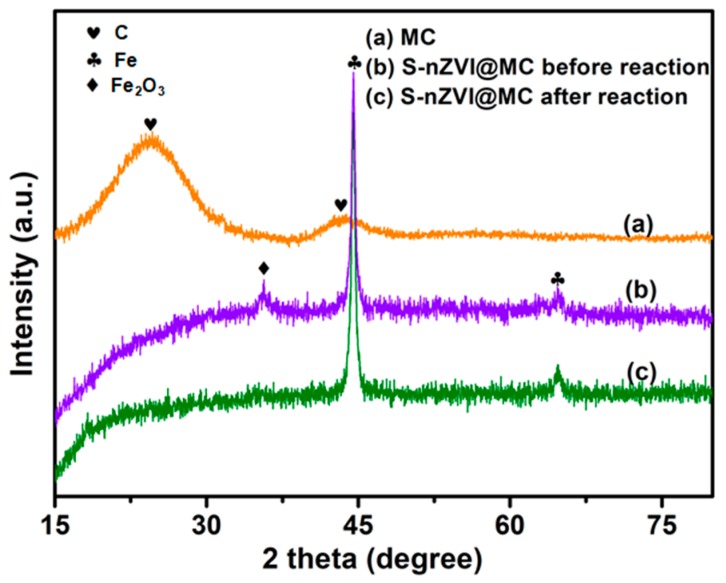
X-ray diffraction (XRD) patterns of MC, S-nZVI@MC before and after reaction.

Figure 3 shows the XRD patterns of S-nZVI@MC and nZVI. The pattern of MC shows two diffraction peaks at 2θ value of 23° and 43°, corresponding to (002) and (101) of the pseudographitic domains, respectively [23]. The diffraction peaks of MC are broad and weak, indicating the limited graphitization degree. In addition, there are no obvious impurity peaks in the XRD pattern of MC, and the content of Si is 0.39 at% according to the XPS result. The above results indicate that the silica template was removed by HF, and the MC was successfully prepared. An obvious diffraction peak appears at 2θ value of 45° and 65° on the patterns of nZVI@MC, which corresponds to the (100) and (200) direction of α-Fe, indicating that the nZVI was successfully loaded on the surface of MC by liquid phase reduction method [24]. The diffraction peak of S-nZVI@MC is sharper, indicating that sulfidation can enhance the crystallinity of nZVI. There is no peak of sulfur species (FeS or FeS_2_) on the XRD pattern of S-nZVI@MC, which may be due to the low sulfur content or poor crystallinity of sulfur species [25]. After 3 h of reaction with TCP, the peak intensity of S-nZVI@MC has no obvious decrease. However, a weaker diffraction peak of Fe_2_O_3_ was observed, indicating that only part of the nZVI reacted with TCP. In addition, the reaction between such non-target reactants as H_2_O and nZVI should be effectively inhibited, which is attributed to the protection effect of FeS [25].

**Figure 4:**
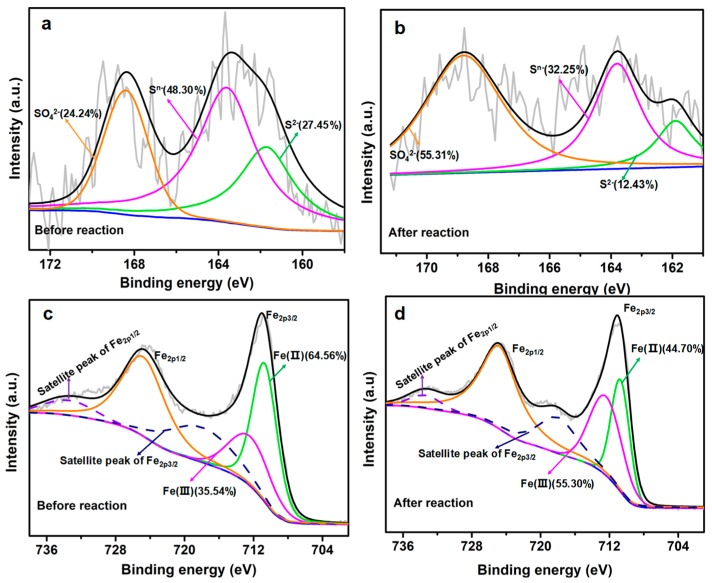
X-ray photoelectron spectroscopy (XPS) spectra of (**a**,**b**) S2p and (**c**,**d**) Fe2p region S-nZVI@MC before and after reaction.

In order to further verify the formation of Fe and S before and after the reaction with TCP, XPS spectra were further analyzed. The results (Figure 4a) showed that the S spectrum can be divided into three peaks, which are located at 161.9 eV, 163.8, and 168.8 eV assigned to S^2−^, S^n−^ and SO_4_^2−^, respectively [26]. As calculated, the molar content of S^2−^, S^n−^ and SO_4_^2−^ is 27.45%, 48.30% and 24.24%. After the reaction with TCP, the peak intensity of S^2−^ decreased obviously, accompany with the increase of S^n−^ (Figure 4b). The S^2−^ molar content decreased to 12.43%, and the molar content of SO_4_^2−^ increased to 55.31%, which indicates that S is oxidized after the reaction with TCP.

The high-resolution XPS spectra of Fe 2p region was shown in Figure 4c,d. The Fe spectrum can be divided into four peaks. The peaks at 710.7 and 712.4 eV are assigned to Fe_2p3/2_ for Fe (II) and Fe (III), respectively [26]. The peak located at 724.8 eV related to Fe_2p1/2_ for Fe (III). Besides, the satellite peak positions for Fe_2p1/2_ and Fe_2p3/2_ were 718 and 733.4 eV. Before reaction, two broad peaks at 724.8 eV (Fe_2p1/2_) and 711 eV (Fe_2p2/3_) corresponding to iron oxides (Fe^2+^ and Fe^3+^) are obvious (Figure 4c), because a portion of nZVI could be oxidized after the reaction with SO_4_^2−^ during preparation or testing process, resulting in the formation of iron oxides or iron sulfides. However, it is worth noting that XPS can only detect a material surface of several nanometers’ depth, and the Fe^0^ core may not be effectively detected [25]. The content of Fe (II) and Fe (III) is 64.56% and 35.54% before reaction. However, the Fe_2p3/2_ peak for Fe (III) is strengthened, while the peak corresponding to Fe (II) is weakened (Figure 4d). The ratio of Fe (II) is decreased to 44.70%, while Fe (III) is increased to 55.30%, which indicates that Fe (0) and part of the Fe (II) in S-nZVI@MC were transformed to Fe (III) after reacting with TCP.

### 3.2. Adsorption Performance

**Figure 5:**
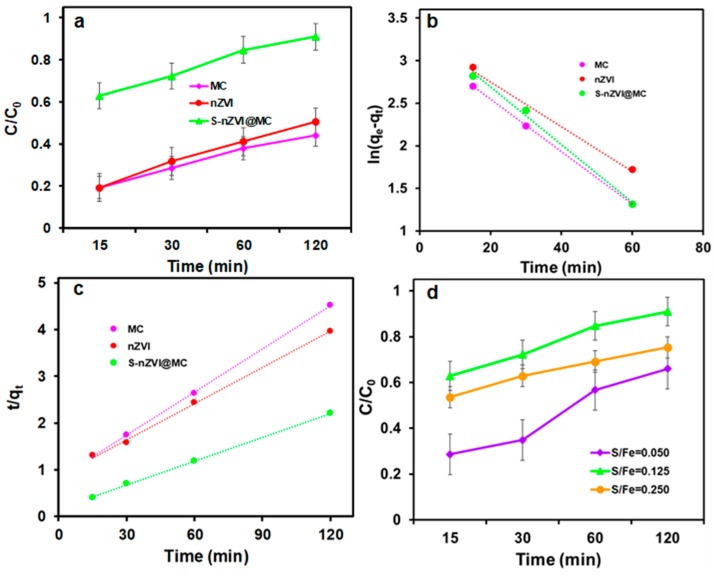
(**a**) The 2,4,6-trichlorphenol (TCP) removal performance, (**b**,**c**) kinetics study by MC, nZVI (**d**) S-nZVI@MC and the effect of Fe/S ratio on removal performance of TCP by S-nZVI@MC.

The removal of TCP by nZVI before and after sulfidation was investigated. As shown in Figure 5a, the MC supporter has a certain adsorption capacity for TCP in water and the removal efficiency is about 30% after 120 min, which can be ascribed to the pore-filling effect. The abundant mesopores can provide accessible channels for TCP molecular transport and adsorption. The removal efficiency of bare nZVI towards TCP was about 50% due to the adsorption and reduction effects. It is noteworthy that the removal efficiency was significantly increased after sulfidation, which is increased to 91%. The enhanced removal efficiency indicated that the sulfide modification greatly increases the reactivity of nZVI to TCP, because the -Cl groups on TCP have strong affinity with S-nZVI due to the hydrophobic effect of iron sulfide. Furthermore, the iron sulfide coated on the surface of nZVI can inhibit the formation of iron oxide, while iron sulfide with low-band gap can promote the rapid transfer of electrons from the nZVI core [27].

The adsorption kinetics of MC, nZVI and S-nZVI@MC were further investigated as the following equations:

pseudo-first kinetic: ln(q_e_ − q_t_) = ln q_e_ − *k*_1_ t (2)

pseudo-second kinetic: t/q_t_ = 1/*k*_2_ q_e_^2^ + t/q_e_ (3)

where q_e_ and q_t_ (mg g^−1^) was the removal capacity of TCP at time t (min) and equilibrium, respectively. *k*_1_ (min^−1^) and *k*_2_ (g mg^−1^ min^−1^) was the equilibrium rate constant of both kinetic models [28,29].

The kinetic fitting results were shown in Figure 5b,c and the kinetics parameters are summarized in Table 1. On basic of the R^2^, the pseudo-second kinetic model can better describe the TCP removal behavior, suggesting that the TCP removal by nZVI and S-nZVI@MC is controlled by chemical processes, and the electronic exchange existed between TCP and S-nZVI@MC. Obviously, the rate constant *k*_2_ of S-nZVI@MC was 0.0018 g mg^−1^ min^−1^, higher than that of MC and nZVI (0.0012 and 0.0007 g mg^−1^ min^−1^), which further reveals a higher affinity between S-nZVI@MC and TCP owing to the hydrophobic effect [28]. The highest *k* of S-nZVI@MC further indicates that the sulfide treatment can increase the reactivity.

Fe/S is one of the most critical factors that would influence the reactivity of S-nZVI@MC. The S-nZVI@MC particles sysnthesized at different S/Fe were further used for TCP removal, and the results are presented in Figure 5d. Obviously, the removal efficiency ranges from 60% to 90% depending on the different S/Fe ratio. However, the removal efficiency of S-nZVI@MC to TCP varies with the S/Fe ratio, for optimal S/Fe (0.125) the maximum removal efficiency can reach 90%. With lower S/Fe (0.05), the lower content of S^2-^ results in ineffective protection of nZVI, and thus the removal efficiency of TCP is lower. The removal efficiency decreased when the S/Fe further increased to 0.25, because excessive iron sulfide, especially FeS_2_ with a higher band gap (0.95) generated could slow down electron transfer [27]. Moreover, the corrosion of zero-valent iron is inhibited at high S/Fe, leading to nZVI reactivity waste. Therefore, it is critical to optimize S/Fe ratio during the sulfidation process, and the optimal S/Fe molar ratio is 0.125 was used in the following TCP removal experiments.

**Figure 6:**
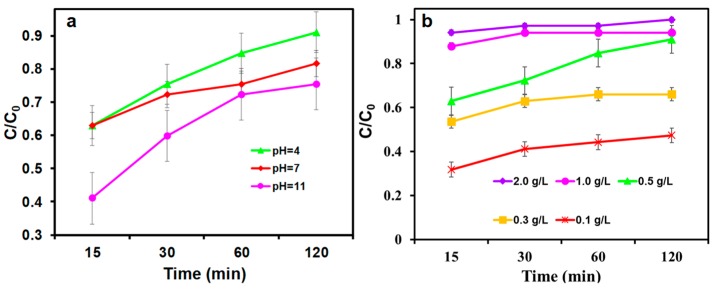
(**a**) Initial pH and (**b**) dosage on removal performance of TCP by S-nZVI@MC.

As known, the pH value of aqueous solution has great effect on nZVI. In acidic conditions, nZVI could be rapidly corroded and tends to exhibit better activity. While in alkaline conditions, a large hydroxide passivation layer is formed on the surface of zero-valent iron, thereby inhibiting its activity. It has been demonstrated that sulfidation modification can promote electron transfer and inhibits surface passivation. However, the effect of sulfidation on nZVI reactivity under different pH conditions still needs further discussion. Herein, the removal efficiency of S-nZVI@MC under different pH value ranging from 4 to 11 was investigated. As shown in Figure 6a, the removal efficiency reached a maximum of 91% at pH of 4.0, and then decreased to 82% when the pH value increased to 11.0, which might be due to the formation of iron oxides on the surface of S-nZVI@BC [30].

The effect of S-nZVI@MC dosage was further investigated under the condition of pH = 4.0, 30 mg L^−1^ TCP aqueous solution and the results are shown in Figure 6b. The removal efficiency of TCP was enhanced when the dosage was increased. Accordingly, the removal efficiency is 57%, 69%, 88%, 91%, 97% and 100% for 0.1, 0.3, 0.5, 1.0, 2.0 and 3.0 g L^−1^, respectively. When the dosage is high, there are more surface reactive and adsorptive sites in the reaction system, so the removal rate of TCP is higher. However, an excessive dosage is likely to cause agglomeration of materials and low utilization of reaction sites. Hence, the dosage of the further study is 0.5 g L^−1^ for TCP removal.

**Figure 7:**
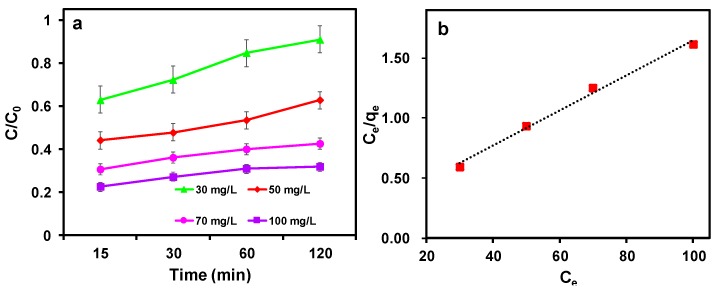
(**a**) Effect of initial concentration on removal performance and (**b**) the linear Langmuir isotherm plot for TCP on S-nZVI@MC.

Figure 7a shows the removal efficiency of S-nZVI@MC towards TCP under different initial concentrations ranging from 30 to 100 mg L^−1^. Generally, the TCP removal efficiency is decreased when the initial concentration increased. As the initial concentration increased from 30 to 100 mg L^−1^, the removal efficiency decreased from 91% to 32%. With higher initial concentration, the surface of S-nZVI@MC is easier to passivate, and thus the diffusion of contaminants and the electron transfer between nZVI and contaminants are inhibited, resulting in a reduced contaminants removal efficiency. Besides, at high concentrations, a large number of target contaminant molecules compete for limited reaction sites on the surface of the S-nZVI@MC, which also leads to reduced TCP removal efficiency [31]. The adsorption isotherm was used to analyze the interactions between TCP and S-nZVI@MC, which further can be fitted by Langmuir and Freundlich models. As shown in Figure 7b, the TCP removal profile can be described well by the Langmuir model, which has a higher correlation coefficient R^2^ (0.993). In contrast, the Freundlich model has a relative lower R^2^ (0.936). Hence, the adsorption of TCP on the surface of S-nZVI@MC has been dominated by monolayer adsorption [32].

**Figure 8:**
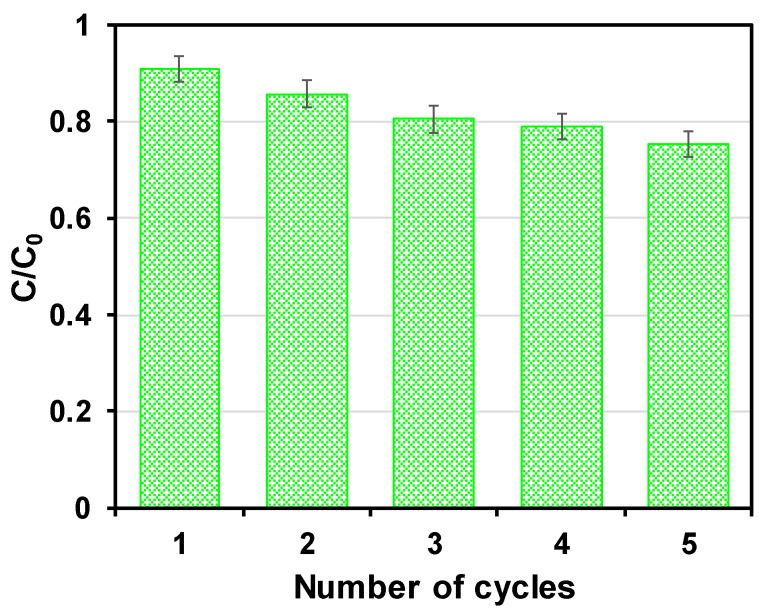
The reuse ability of S-nZVI@MC towards TCP.

Reusability is another critical property for the application of nZVI-based material. The reusability of S-nZVI@MC was further tested in 30 mg L^−1^ TCP solution with the dose of 0.5 g L^−1^ and reaction time of 3 h. As shown in Figure 8, the stability of S-nZVI@MC was evaluated by reactions 5 times. The reaction ability has no significant decline after five repeated experiments, indicating there was no significant passivation on the surface of S-nZVI@MC. The good stability can be ascribed to effective sulfidation. Hence, the results of reusability experiments further demonstrate that S-nZVI@MC can be practically applied to pollutant adsorption and reduction.

## 4. Conclusions

In summary, biomass-derived MC-loaded S-nZVI was successfully prepared for effective TCP removal. MC was selected as the support for nZVI, because its mesoporous structure was beneficial to nZVI dispersion and mass transfer. Moreover, MC prepared from biomass waste was a cost-effective material for practical application. Further surface sulfidation improved the antioxidation of nZVI due to the protection effect of FeS shell. SEM, TEM, XRD and XPS measurements demonstrated that the nZVI particles were successfully loaded on the MC supporter and the FeS was well coated on the surface the nZVI. Furthermore, the aggregation of nZVI and the iron oxide formation was prevented according the structure characterization. S-nZVI@MC was most effective for TCP removal as compared to MC and nZVI, and the removal efficiency reached the highest value at a ratio of S/Fe = 0.125. The effect of ambient conditions such as solution pH, initial concentration and dosage on TCP removal were also studied, and results revealed that the low solution pH value and concentration and higher dosage had positive effect on TCP removal. Furthermore, the removal behavior was fitted with a Langmuir model and pseudo-second kinetic model. The removal mechanism of S-nZVI@MC towards TCP could by analyzed by fast adsorption and effective reduction by S-nZVI. Therefore, the synergistic effect of MC loading and surface sulfide-modification promoted high removal efficiency of S-nZVI@MC towards TCP.

## Figures and Tables

**Table 1:** Kinetic model parameters for the removal of 2,4,6-trichlorphenol (TCP) by MC, nZVI and S-nZVI@MC.

Sample	Pseudo-First-Order-Model			Pseudo-Second-Order-Model		
	*k*_1_ (min^−1^)	q_e_ (mg g^−1^)	r^2^	*k*_2_ (g mg^−1^ min^−1^)	q_e_ (mg g^−1^)	r^2^
MC	0.0308	23.64	1.000	0.0012	32.57	0.9998
nZVI	0.0262	26.43	0.9894	0.0007	38.75	0.9985
S-nZVI@MC	0.0339	29.14	0.9951	0.0018	58.82	0.9996
